# From environmental heterogeneity to stability: Beta‐diversity stabilizes biomass across space

**DOI:** 10.1002/ecy.70498

**Published:** 2026-09-17

**Authors:** Benedikt Brunner, James G. Hagan, Jesper Hassellöv, Julia Mari Murata, Lara Colaço Martins, Pedro de La Patelliere, Elena Schrofner‐Brunner, Lars Gamfeldt

**Affiliations:** ^1^ Department of Marine Sciences, Tjärnö Marine Laboratory University of Gothenburg Strömstad Sweden; ^2^ Community Ecology Lab, bDIV Research Group, Department of Biology Vrije Universiteit Brussel (VUB) Brussels Belgium; ^3^ University of Algarve Faro Portugal; ^4^ NOVA School of Science and Technology (FCT NOVA), Department of Environmental Sciences and Engineering Universidade NOVA de Lisboa Caparica Portugal; ^5^ Department of Marine Sciences University of Gothenburg Gothenburg Sweden; ^6^ Gothenburg Global Biodiversity Centre Gothenburg Sweden; ^7^ Centre for Sea and Society Gothenburg Sweden

**Keywords:** beta‐diversity, biodiversity, biomass stability, ecosystem functioning, environmental heterogeneity, marine, response diversity, spatial insurance, species sorting, turnover

## Abstract

Understanding how biodiversity influences ecosystem stability across heterogeneous landscapes is a central challenge in ecology. The spatial insurance hypothesis proposes that turnover in species composition stabilizes ecosystem processes in heterogeneous environments if species dominate in the habitats to which they are best adapted. Yet, empirical evidence remains mixed, partly due to confounding factors and overlooked causal mechanisms. We combined metacommunity simulations and a field experiment with marine benthic communities to test how β‐diversity (species turnover) affects the spatial stability of biomass (its invariability among sites at a single point in time). The simulations, which varied the strength of selection via niche overlap, revealed that turnover enhanced spatial stability of biomass in scenarios with low to medium niche overlap, but only when environmental heterogeneity was statistically accounted for. This stabilizing effect weakened when species' niches were broader. In our field experiment, high turnover similarly mitigated the negative impact of environmental heterogeneity on spatial stability. Together, these results provide strong support for the spatial insurance hypothesis, demonstrating that species turnover can stabilize ecosystem functioning across heterogeneous landscapes. Crucially, failing to account for environmental heterogeneity can mask this effect, leading to the mistaken conclusion that turnover has a neutral or destabilizing effect. Our study highlights the importance of a causal framework that disentangles the effects of environmental heterogeneity and β‐diversity, helping to resolve previous inconsistencies and advance a mechanistic understanding of biodiversity–stability relationships at landscape scales.

## INTRODUCTION

Understanding how biodiversity shapes ecosystem processes across spatial scales remains a central challenge in ecology (Ali, 2023; Gonzalez et al., 2020; Le Provost et al., 2023; Loreau et al., 2021; Mori et al., 2021). Research has primarily focused on local scales, often intentionally striving to minimize the influence of environmental heterogeneity. Through hundreds of experiments, we have learned that biodiversity loss and associated changes in local species interactions generally have negative consequences for ecosystem processes (Cardinale et al., 2012). However, our knowledge of how biodiversity affects ecosystem processes at larger scales relevant for landscape management is still limited (Gamfeldt et al., 2023; Gonzalez et al., 2020). Because many ecological analyses remain correlational (Franks et al., 2025), we here adopt an explicitly causal framework to separate the role of turnover in biodiversity from the role of environmental heterogeneity.

An increase in spatial scale is generally associated with increased spatial environmental heterogeneity (Hart et al., 2017). Typically, a larger landscape comprises more habitat types and ecological niches, creating a mosaic of environmental conditions across space. As species respond differently to these spatially variable environmental conditions, a large landscape is expected to host more species than a smaller one, with distinct species compositions emerging between different habitat sites (Stein et al., 2014). High environmental heterogeneity is thus often expected to promote high species turnover (i.e., replacement, an aspect of β‐diversity). In the context of ecosystem processes (e.g., biomass production), species' different responses to the environment should ensure stability if different species drive a process in different environments—the spatial insurance hypothesis (Loreau et al., 2003; van der Plas, 2019). It follows that, if β‐diversity is reduced, the spatial stability (invariability) of ecosystem processes should decline. Yet, empirical testing of this prediction is inconclusive (van der Plas et al., 2023). Previous studies have shown mixed results, with some supporting the stabilizing effect of β‐diversity, for example, Chen et al. (2022), while others indicate no relationship, for example, Yang et al. (2022). This discrepancy in empirical findings indicates a gap that may stem from differing analytical approaches and from treating these relationships as correlational, which may obscure direction and magnitude of effects. Here, we clarify mechanisms by quantifying environmental heterogeneity and modeling turnover as a mediating pathway to spatial stability (sensu causal mediation). A key challenge is that environmental heterogeneity is expected to increase turnover (via species sorting) while also directly decreasing spatial stability (by creating among‐site differences in productivity), making heterogeneity a confounder if it is not explicitly accounted for.

Spatial scale can fundamentally alter the balance among the core processes of community assembly: selection, drift, and dispersal (Vellend, 2016). At larger spatial extents or in fragmented landscapes, dispersal limitation (e.g., barriers preventing high‐functioning species from reaching suitable habitats) and demographic stochasticity (drift) can decrease the role of environmental filtering, which is the major cause of species sorting (Catano et al., 2025; Chase, 2010; Hubbell, 2001; Nekola & White, 1999). Dispersal limitation and drift may thus generate patterns of species turnover decoupled from species' niche differences (Leibold et al., 2004; Vellend, 2010). The spatial insurance hypothesis assumes that β‐diversity is mainly the result of selection (i.e., species sorting), where the local environment favors species that have high ecosystem functioning. Selection‐caused turnover is predicted to sort high‐functioning species into their respective environmental optima, thereby enhancing spatial stability of ecosystem processes across heterogeneous landscapes (Loreau et al., 2003). In contrast, turnover driven by drift or dispersal constraints may result in weak links between species traits and environmental conditions, potentially weakening the spatial stability of ecosystem processes (van der Plas et al., 2023). Studies on β‐diversity often conflate turnover caused by selection with that caused by neutral or dispersal‐driven dynamics, likely explaining why empirical tests of spatial insurance remain equivocal (van der Plas et al., 2023). To clarify these scale‐dependent mechanisms, it is important to disentangle how the shifting balance of assembly processes shapes β‐diversity and ecosystem stability. Our study addresses this by testing whether turnover consistent with species sorting (not β‐diversity per se) is important for the spatial stability of ecosystem functioning.

**FIGURE 1:**
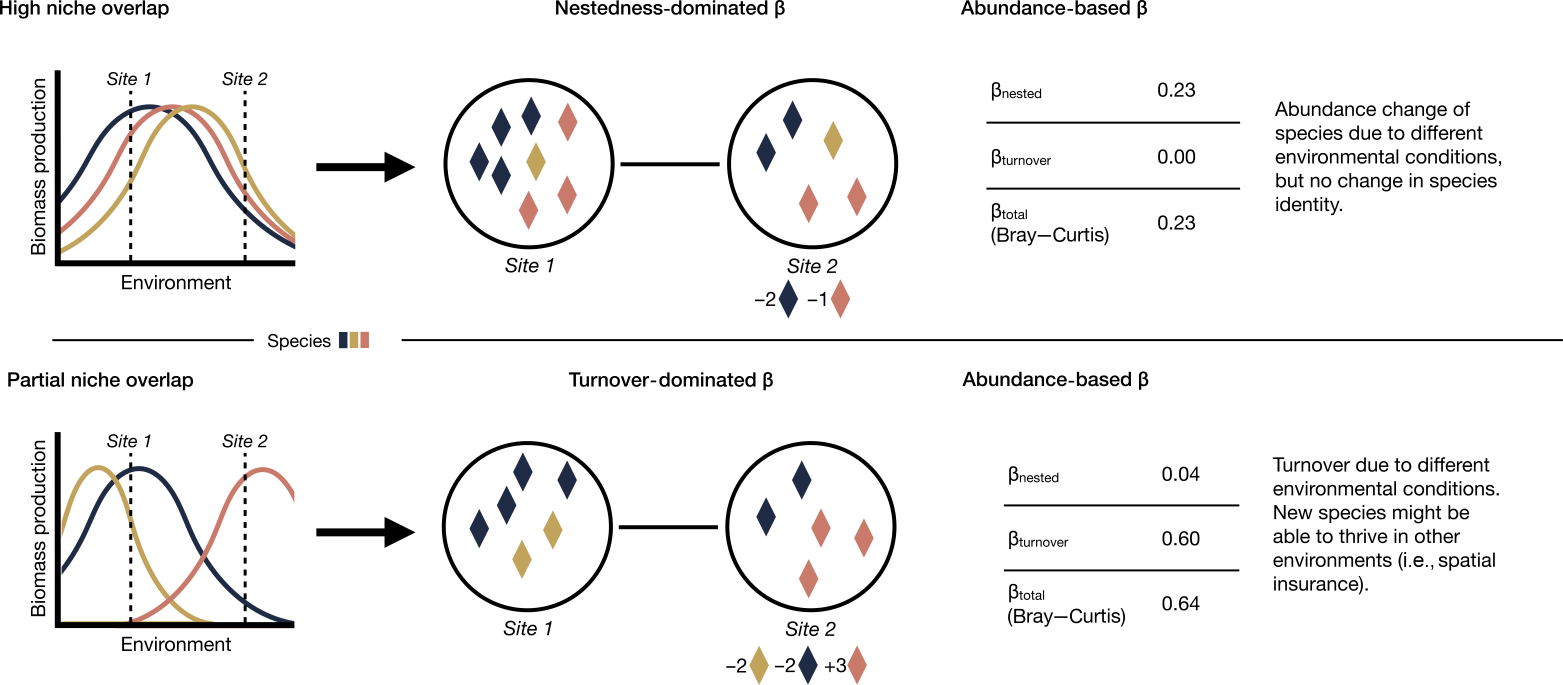
The Bray–Curtis distance shows the abundance‐based total dissimilarity between the two communities. This can be partitioned into its turnover component (balanced variation in abundance between sites) and nestedness component (unidirectional abundance gradients). Similar patterns of species distribution could arise through drift and dispersal.

Spatial β‐diversity measures the difference in species composition between different sites in a metacommunity (Anderson et al., 2011; Whittaker, 1960). To better isolate mechanisms, these compositional differences can be separated into two components—nestedness and turnover—which are driven by different mechanisms (Baselga, 2010; see Figure 1). Nestedness captures abundance gradients and richness differences, whereby assemblages at one site can form subsets of another. Turnover defines the replacement of species between sites, often driven by environmental filtering and niche differences (Vellend, 2016). While both components contribute to overall β‐diversity, turnover is the component most directly linked to the niche‐based mechanisms underlying the spatial insurance hypothesis. Partitioning β‐diversity into these two components allows us to disentangle the relative contributions of nestedness and turnover to overall community variation (Baselga, 2010, 2013). This approach can significantly improve our understanding of the relationship between community change and ecosystem processes by highlighting the mechanisms driving biodiversity patterns (Mori et al., 2018; Omidipour et al., 2021).

Spatial stability, in the context of this study, refers to the spatial invariability of biomass among sites at a given sampling date. For each site pair, we quantify spatial stability as the ratio of the smaller to the larger biomass value, a bounded measure closely related to the inverse of a spatial coefficient of variation (CV) used in other strictly spatial applications of stability (e.g., Daleo et al., 2023; Eisenhauer et al., 2011; Weigelt et al., 2008). Because our focus is on purely spatial differences in ecosystem functioning at a single point in time, this ratio provides a direct spatial stability metric that does not require temporal replication.

**FIGURE 2:**
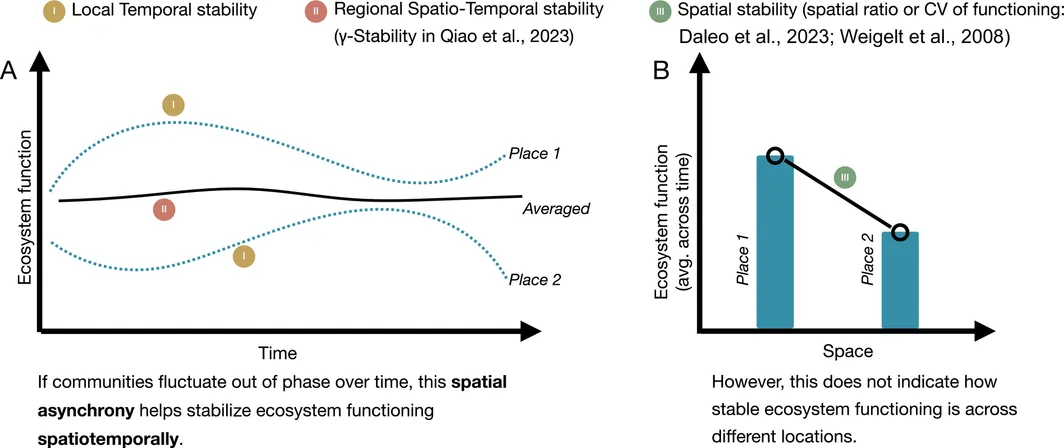
(A) Ecosystem functioning (e.g., biomass production) fluctuates out of phase at two places over time (I: Local temporal stability). Even though local stability is weak, regional spatiotemporal stability (II) is high, and the combination of I and II leads to high spatial asynchrony (sensu Qiao et al., 2023). Spatial asynchrony can stabilize community responses spatiotemporally, as one place might have higher functioning while another has decreased functioning. (B) However, spatial asynchrony does not indicate how variable ecosystem functioning is across locations (i.e., comparing the average functioning of both places, III).

The term “stability” often carries a temporal connotation, because ecological stability is commonly assessed through temporal variability in ecosystem properties (Donohue et al., 2016; Gonzalez et al., 2020). Spatial asynchrony extends this temporal framework by describing how out‐of‐phase temporal fluctuations among sites can stabilize aggregate ecosystem functioning over time (Loreau et al., 2003; Loreau & de Mazancourt, 2013; Qiao et al., 2023; Wang et al., 2021, 2024). However, spatial asynchrony is a function of temporal covariances among sites and therefore does not quantify how evenly ecosystem functioning is distributed across space (i.e., among‐site differences in mean biomass at a given time). Moreover, asynchrony is difficult to estimate or interpret when ecosystem functioning is measured at a single time point, when local communities show little temporal variation, or when sites respond synchronously to environmental drivers (e.g., a uniform growing season). As a result, even with high spatial asynchrony, sites may still differ substantially in their mean functioning levels (illustrated in Figure 2A,B). Since we are interested in the consistency of ecosystem functioning in heterogeneous landscapes, we thus define spatial stability as the invariability of functioning across space.

**FIGURE 3:**
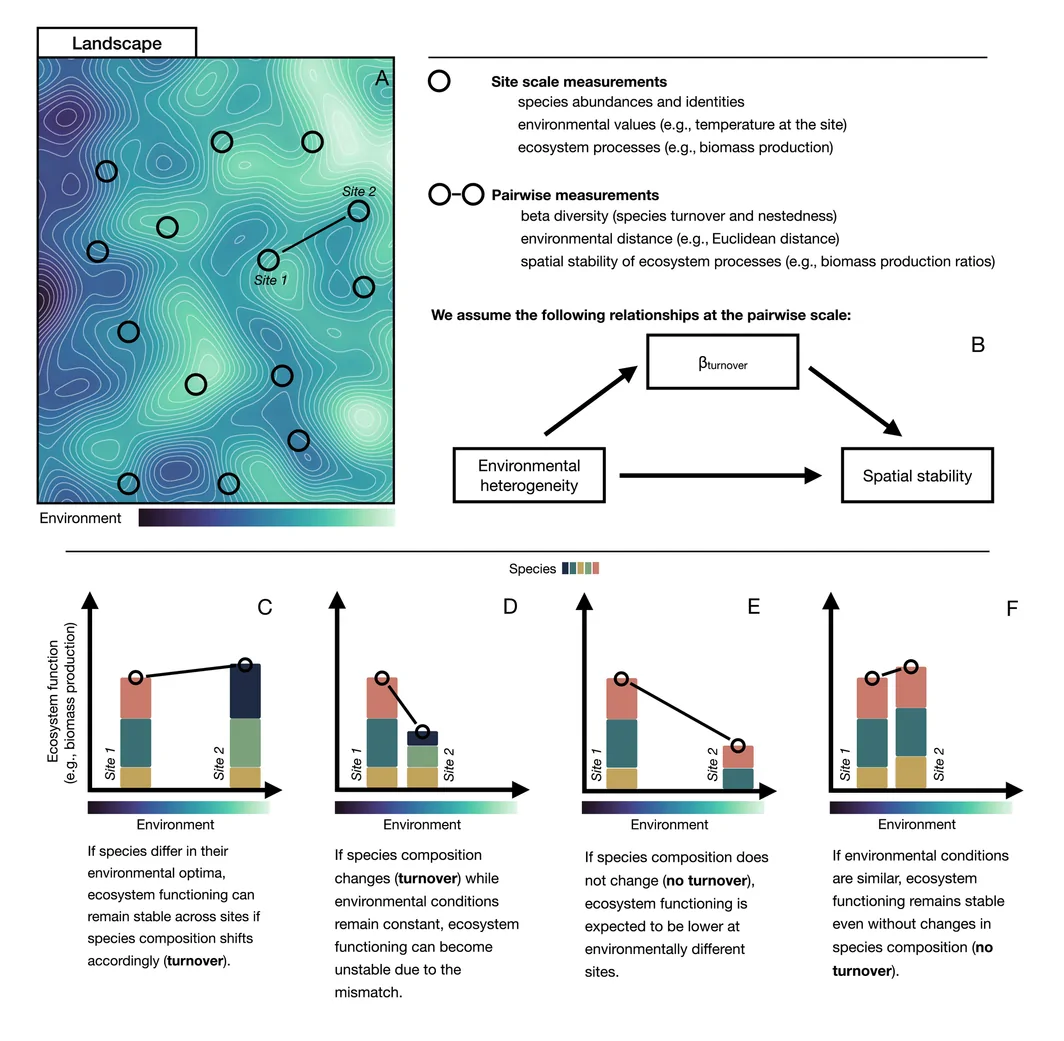
The effects of biodiversity on ecosystem stability can be measured at different scales. (A) A metacommunity consists of multiple sites, which can be aggregated in pairs to measure how β‐diversity influences the spatial stability of ecosystem processes (e.g., biomass production) in the landscape. These sites differ in species identity and abundance, environmental conditions, and baseline levels of ecosystem processes. (B) A conceptual model illustrating the relationship between β‐diversity (species turnover), environmental heterogeneity, and the spatial stability of ecosystem processes. To accurately assess the role of β‐diversity, environmental heterogeneity must be accounted for statistically or experimentally, because the effect of β‐diversity depends on environmental heterogeneity. (C) High turnover combined with high environmental heterogeneity can maintain high spatial stability in ecosystem processes. (D) High turnover under constant environmental conditions can lower spatial stability due to a mismatch between community composition and environmental conditions. (E) Low turnover under high environmental heterogeneity reduces spatial stability across sites. (F) Low turnover under low environmental heterogeneity results in high spatial stability in ecosystem processes.

In line with the insurance hypothesis, we predict that in landscapes characterized by significant environmental heterogeneity, high spatial turnover, driven by species sorting, will facilitate spatial stability of biomass production (which we use as our estimate of ecosystem functioning). This prediction only holds if species have different environmental optima (niches) where they are the superior competitors and their productivity is maximized (Loreau et al., 2021). When different species thrive under different conditions, they maintain biomass production at the landscape scale even though species composition varies across sites. Conversely, turnover caused by ecological drift or dispersal, such as the presence of sink populations or invasive species (Gilbert & Levine, 2017), could decrease production by introducing species that are less adapted to local environmental conditions (Vellend, 2016). Moreover, differences among local sites in resource availability and environmental conditions can directly decrease the spatial stability of biomass production. Productivity will be high at some sites and low in others, thereby causing high variation across the landscape. Because such differences may covary with the effect of β‐diversity on spatial stability, failing to separate them out could introduce omitted variable bias, leading to an inaccurate estimation of β‐diversity's impact on biomass production stability (Byrnes & Dee, 2025; Rinella et al., 2020; see Figure 3A–F).

We apply a causal framework for the spatial stability of biomass production with β‐diversity serving as a mediator (Figure 3B). Specifically, we measure environmental heterogeneity, β‐diversity, and spatial stability at the same hierarchical level across the landscape (pairs of sites, Figure 3A). This integrated approach enables us to disentangle the direct influence of β‐diversity on spatial stability from the confounding effects of environmental heterogeneity, thereby minimizing omitted variable bias. It is our aim to potentially elucidate some of the inconsistencies observed in previous empirical studies (see van der Plas et al., 2023) and contribute to a more robust foundation for predicting how changes in environmental heterogeneity and β‐diversity affect the spatial stability of biomass production at larger spatial scales.

We test the following three hypotheses:

1. High spatial turnover in species composition increases the spatial stability of biomass production among local sites in heterogeneous environments.
2. The spatially stabilizing effect of species turnover is strongest when niche overlap is low and environmental heterogeneity is high.
3. Accounting for environmental heterogeneity is essential for accurately estimating the turnover–stability relationship; omitting it biases the inferred effect of turnover.

We first employed metacommunity models (Thompson et al., 2020) to simulate scenarios with varying species niche widths. This approach allowed us to investigate how niche overlap influences the turnover–stability relationship in spatially heterogeneous environments. We then conducted a field experiment with marine benthic organisms—a system with strong environmental filters and high dispersal—to test the predictions from our models in a natural ecosystem. In this paper, we focus exclusively on spatial stability.

## METHODS

### Metacommunity simulations

We simulated metacommunities using a process‐based discrete‐time Beverton–Holt metacommunity model (Thompson et al., 2020) implemented in R (package mcomsimr). For species *i* in site *x* at the time *t*, abundances were computed as follows:

$$N_{\mathrm{ix}}\left(t+1\right)=\frac{N_{\mathrm{ix}}\left(t\right)\;r_{\mathrm{ix}}\left(t\right)}{1+\sum_{j=1}^{S}\alpha_{\mathrm{ij}}\;N_{\mathrm{jx}}\left(t\right)}+I_{\mathrm{ix}}\left(t\right)-E_{\mathrm{ix}}\left(t\right),$$

where *r*_*ix*_(*t*) is the density‐independent abiotic response (Gaussian niche) to the site environment, α_*ij*_ are competition coefficients governing local density dependence, and *I*_*ix*_(*t*) and *E*_*ix*_(*t*) represent immigration and emigration among sites (distance‐decay dispersal kernel; Appendix S1: Section S1). Each metacommunity comprised 50 sites and 20 species. Site environments were drawn from a uniform distribution *U*(0,1) and held constant through time within a site, and species' environmental optima were evenly spaced along the gradient (0–1). We evaluated four niche‐breadth scenarios (Table 1); species‐specific maximum growth rates were drawn from a Poisson distribution to allow for rare high growth rates. For the main simulations, each replicate included an initialization phase (100 timesteps) and a burn‐in phase (100 timesteps), followed by 100 analyzed timesteps (total 300 timesteps). A stable equilibrium was usually reached after the burn‐in phase. We retained abundances from the final analyzed timestep for downstream pairwise calculations of turnover, environmental heterogeneity, and spatial stability (Appendix S1: Section S4). To make sure our results are not biased by our choice of sampling time, we provide a sensitivity analysis that evaluates the implications of sampling early versus late in our simulations rather than assuming equilibrium (Appendix S1: Section S5). An illustrative example of species' abiotic responses and transient dynamics across the gradient is shown in Appendix S1: Figure S5.

**TABLE 1:** Four (A–D) scenarios for metacommunity simulations.

ID	Scenario	Niche breadth	No. simulations
A	Low niche overlap	0.1	100
B	Medium niche overlap	0.2	100
C	High niche overlap	0.5	100
D	Complete niche overlap	5.0	100

*Note*: The niche breadth is defined as the SD of the Gaussian response to the environment (environment ranges from 0 to 1). Larger niche breadths result in a higher niche overlap.

### Experiment with marine benthic communities

The experiment was conducted between 7 July and 5 September 2022 in the Tjärnö archipelago on the Swedish west coast (58°52′30.0″ N, 11°08′42.0″ E). This archipelago is a shallow coastal environment situated in the northeastern arm of the Skagerrak, characterized by rocky shores, sandy beaches, and mudflats. The marine environment has a variable salinity, generally ranging between around 15–30 practical salinity units, and moderate water temperatures (ca. 15–20°C) during summer. Local hydrodynamic conditions create spatial heterogeneity in turbidity, wave exposure, and water flow, supporting a diverse benthic community typical of hard‐bottom substrates (Berntsson & Jonsson, 2003). Common sessile and sedentary animal species include barnacles (*Amphibalanus improvisus*), bryozoans (*Electra pilosa*, *Membranipora membranacea*, *Callopora rylandi*), ascidians (*Ascidiella scabra*, *Ciona intestinalis*), and hydrozoans (*Clytia* sp., *Laomedea flexuosa*). These organisms frequently colonize artificial structures such as boat hulls in addition to natural rocky habitats (Berntsson & Jonsson, 2003).

**FIGURE 4:**
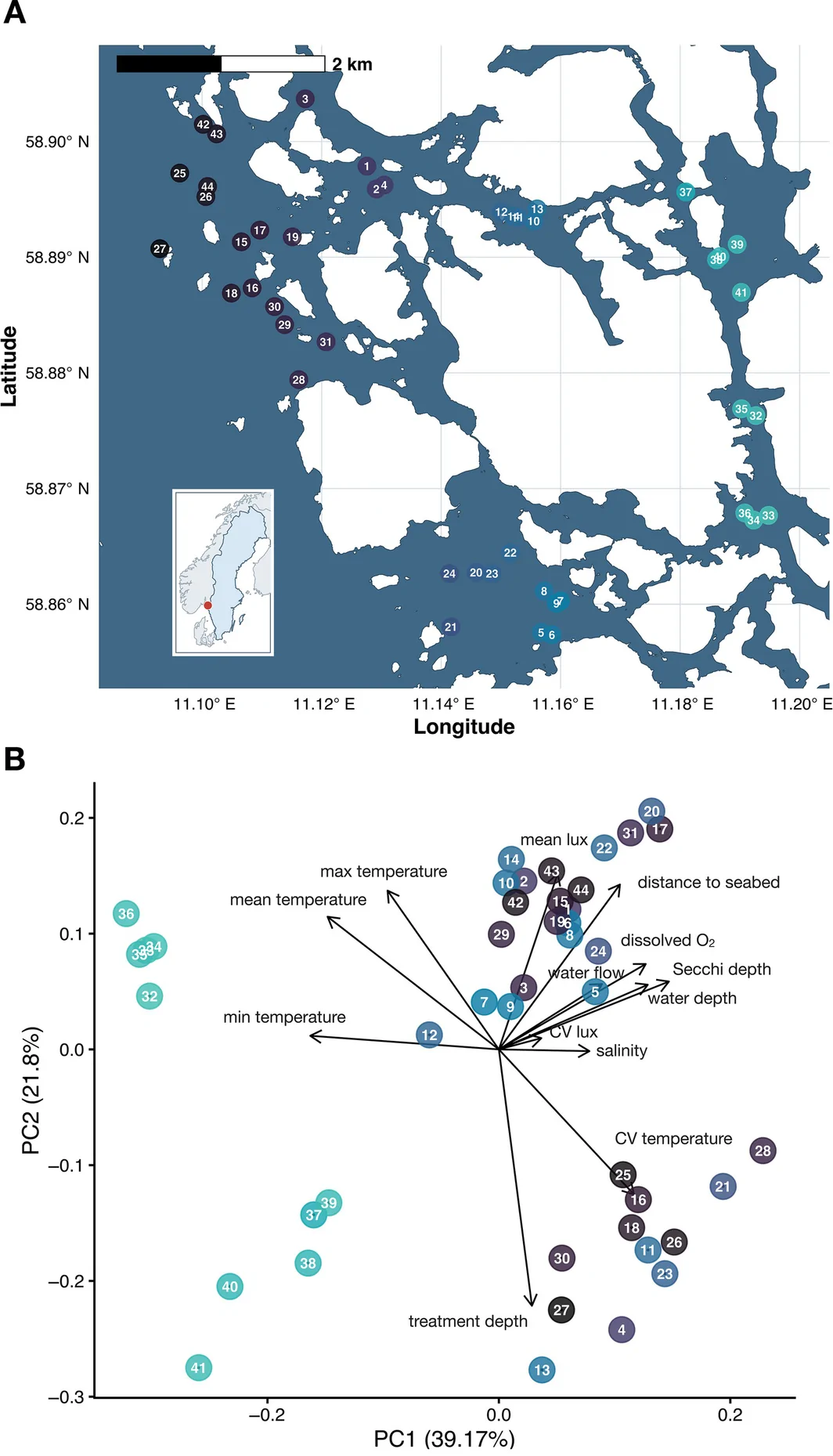
(A) Map of the experimental sites, with the inset showing the location of our study region in Sweden. By the first sampling time point (out of three), six sites were lost due to buoy detachment, reducing the number of sites from 50 to 44. (B) Principal components analysis (PCA) of environmental variables (CV, coefficient of variation of observed values; max, maximum observed value; min, minimum observed value). The closer the points are on the PCA, the more similar their environments are. Colors indicate longitude.

A total of 150 polymethyl methacrylate (PMMA) panels (10 × 11 cm) were deployed across 50 sites (Figure 4A). These sites were selected based on a priori geographical data to cover a large portion of the environmental gradient in the archipelago. Environmental variables included water depth, turbidity, and wave exposure, with a minimum distance of 50 m between sites. The exact positions of the sites were randomized. Settling panels were mounted on rigid frames suspended from surface buoys, maintaining a constant distance to the water surface of 3 or 6 m at all sites. The two depth strata were used to increase environmental heterogeneity among sites with otherwise similar exposure and temperature. Each site received three settling panels, with additional panels for a different experiment. The panels were destructively sampled sequentially after 34, 47, and 60 days, with one panel collected at each sampling point. During the experiment, six sites were lost due to buoy detachment, reducing the number of sites from 50 to 44 prior to the first time point (Figure 4A). Another site was lost between the second and third time point, decreasing the number to 43. Environmental data were measured at each site and included the depth of the settling panels, the distance from the panels to the seabed, and the overall depth (distance from the surface to the seabed), water temperature (recorded hourly, with mean, minimum, maximum, and CV), light intensity (hourly, with mean and CV), salinity, oxygen concentration, Secchi depth for water clarity, and water flow. Water flow was approximated at three time points using gypsum block dissolution over approximately 30 h (Muus, 1968; Porter et al., 2000). Temperature and light were measured by deploying one light/temperature logger at each site (Onset MX2202 HOBO Pendant and Onset UA‐002‐64 HOBO Pendant).

Benthic organisms from the surrounding environment naturally settled on the panels (Appendix S1: Figure S1). Organisms were identified visually to the highest taxonomic resolution feasible and grouped into five operational taxonomic units (OTUs): barnacles (mostly *Am. improvisus*), bryozoans (*E. pilosa*, *M. membranacea*, *Ca. rylandi*), *As. scabra*, hydrozoans (*Clytia* sp., *L. flexuosa*), and *Ci. intestinalis*. After sampling, organisms were carefully scraped off the panels, sorted by OTU, and weighed to assess wet, dry, and ash‐free dry masses. To obtain dry masses, the organisms were dried in a drying oven at 60°C for 48 h. Ash‐free dry masses were determined by combusting the dry biomass at 500°C in a muffle furnace and subtracting the remaining ash mass from the dry mass, providing a reliable measure of organic matter and biomass energy content (Weil et al., 2019).

To quantify environmental heterogeneity between the sites, principal components analysis (PCA) was conducted using all measured environmental variables (Figure 4B), excluding those used for a priori site selection. We summarized correlated environmental variables with PCA and computed Euclidean distances in the first three PC axes (PC1: 39.2%, PC2: 21.8%, PC3: 10%; 71% cumulative) to minimize collinearity and emphasize dominant environmental gradients relevant to assembly. We computed Euclidean distances between sites in PC1–PC3; these distances were our pairwise heterogeneity metric. Each sampling point (at Days 34, 47, and 60) was analyzed independently, as the focus of this study was on purely spatial differences rather than on temporal dynamics.

To visualize species' realized environmental niches, we fitted generalized additive models (GAMs) for each OTU with ash‐free dry mass as the response and environmental PC1–PC3 as predictors. A hurdle approach was used to account for zero‐inflated biomass (binomial presence model combined with a Tweedie biomass model). Expected ash‐free dry masses were predicted across PC1 and PC2 while holding PC3 at its median and shown as heatmaps.

### Data analysis

All statistical analyses were conducted using R version 4.4.2 (R Core Team, 2024). We investigated the relationships among spatial stability (i.e., invariability) of biomass, turnover, and environmental heterogeneity in both simulated and experimental metacommunities using the same statistical methods. We computed pairwise measures for all possible pairs of sites within each metacommunity to assess how differences in community composition and environmental conditions relate to spatial stability.

To derive these pairwise measures, we employed a distance‐based approach using the upper triangle of the distance matrix. By restricting our analysis to the upper triangle, we ensured that each pair of sites was only included once, thereby avoiding redundancy in the data. This method provided us with 1225 unique site pairings for the simulated metacommunities (with 50 sites) and 903 or 946 pairings for the experimental metacommunities (with 43 or 44 sites, respectively).

Because individual sites were represented in multiple pairwise measures, our approach introduced pseudoreplication (Arnqvist, 2020). We addressed this issue by incorporating Bayesian mixed‐effects regression models with multi‐membership random effects (Bürkner, 2018; Park & Beretvas, 2020; Wurz et al., 2025). This modeling strategy accounts for the nonindependence of observations, as each pair involves two sites that may appear in other comparisons, ensuring that our parameter estimates, uncertainty of estimates, and inference on the relationships among spatial biomass stability, β‐diversity, and environmental heterogeneity remain robust. We fitted separate models for each time point.

#### β‐diversity and turnover

We calculated the balanced variation component of the Bray–Curtis dissimilarity (i.e., the abundance‐based turnover component) for each pair using the *betapart* package in R (Baselga et al., 2023). This metric quantifies the extent of species turnover between two sites while considering species abundances, thereby capturing changes in community composition attributable to species replacement rather than differences in total abundance. The partitioning of the Bray–Curtis dissimilarity *d*_BC_ is given by Equation (1).

(1) $$d_{\mathrm{BC}}=d_{\mathrm{BC}\;\text{balanced variation}}+d_{\mathrm{BC}\;\text{abundance gradient}}$$

where *d*_BC balanced variation_ is the balanced variation (turnover) component and *d*_BC abundance gradient_ is the abundance gradient (nestedness) component (Baselga, 2013). In our analysis, we focused on *d*_BC balanced variation_ to directly assess species turnover (Anderson et al., 2011).

We selected this abundance‐based turnover metric for two primary reasons. First, by separating species replacement from overall abundance differences, we avoid confounding the effect of turnover with simple gains or losses in total community abundance, aligning better with the mechanisms of the spatial insurance hypothesis. Second, unlike metrics based on presence/absence data (e.g., the Jaccard index), *d*_BC balanced variation_ utilizes species abundance information, making it more sensitive and appropriate for detecting compositional turnover where changes in relative abundance are significant while species identities overlap largely between places.

#### Environmental heterogeneity

Environmental heterogeneity between sites was quantified using pairwise Euclidean distance in PCA space. For the experimental data, we conducted a PCA on the measured environmental variables (excluding those used for initial site selection) to reduce dimensionality and identify the main environmental gradients. The first three principal components (PC1: 39.2%; PC2: 21.8%; PC3: 10%) were retained as they captured the majority of environmental variation. We then calculated the Euclidean distances between sites in this reduced environmental space using the *vegan* package (Oksanen et al., 2024). This approach ensures our heterogeneity metric reflects the dominant environmental gradients driving community structure across the archipelago. These distances served as our measure of environmental heterogeneity for each pair. For the simulated metacommunities, each site was assigned a single environmental value ranging from 0 to 1. We calculated environmental heterogeneity between sites using the same Euclidean distance function. In this univariate case, the Euclidean distance is equivalent to the absolute environmental difference between two sites.

#### Spatial stability of biomass

We quantified spatial stability (spatial invariability) as the ratio of the smaller to the larger biomass value in each site pair. For each site, total biomass was calculated by summing the abundances (in simulations) or the ash‐free dry masses (in the experiment) of all species present. Spatial stability was computed using Equation (2).

(2) $$\text{Spatial stability}=\frac{\min\left(B_{i},B_{j}\right)}{\max\left(B_{i},B_{j}\right)}$$

where *B*_*i*_ and *B*_*j*_ represent the total biomass at site *i* and site *j*, respectively. This metric reflects variability in biomass between sites and is bounded between 0 and 1 (no values were exactly 0 or 1). The metric is strongly related to the inverse of the CV (Wang & Loreau, 2014, 2016). A higher value indicates lower spatial variability in total biomass between sites, reflecting greater stability of ecosystem processes at the regional scale at a given focal time point. Distributions of turnover, environmental heterogeneity, and spatial stability across datasets are shown in Appendix S1: Figure S4.

#### Statistical modeling

To investigate relationships among spatial stability, environmental heterogeneity, and turnover, we employed Bayesian mixed‐effects regression models using the brms package in R (Bürkner, 2018). All continuous predictors were standardized (z‐transformed) to a mean of 0 and a SD of 1, facilitating convergence and comparability of effect sizes.

Because our response variable, *spatial stability*, is continuous and constrained between 0 and 1, we employed a beta regression with a logit link to capture its distribution appropriately.

We fitted two main models for each of the three time points:

1. *Full model*:
  spatial stability ~ turnover × environmental heterogeneity + (turnover × environmental heterogeneity|mm(site_*i*_, site_*j*_))
  This model evaluates the interactive effects of turnover and environmental heterogeneity on spatial stability. The multi‐membership random effects structure, indicated by mm(site_*i*_, site_*j*_), accounts for the fact that each observation involves a pair of sites (site_*i*_ and site_*j*_), which can appear in multiple pairwise comparisons.
2. *Reduced (confounded) model*:
  spatial stability ~ turnover + (turnover|mm(site_*i*_, site_*j*_))
  This model assesses the effect of turnover on spatial stability without including environmental heterogeneity.

For both models, we did not specify custom priors; therefore, priors follow the default brms priors (Bürkner, 2018). Pairwise comparisons involve two sites (site_*i*_ and site_*j*_) that can appear in multiple pairs, which introduces pseudoreplication if treated as independent observations. To address this, we specified a multi‐membership random effects structure using the mm() function. This approach assigns random intercepts and slopes to both sites within each pair, thereby accounting for the nonindependence inherent in our pairwise data (Browne et al., 2001; Park & Beretvas, 2020). Posterior predictive checks and leave‐one‐out cross‐validation (LOO) were conducted to assess model fit and verify that the models adequately captured the underlying structure of the data (Vehtari et al., 2017). Additionally, we analyzed other regression paths (e.g., stability ~ environmental heterogeneity) using the same method for a complete mediation analysis.

#### Average marginal effects and confounding bias

Because turnover effects are context‐dependent in the full model (turnover × environmental heterogeneity) and regression coefficients are on the link scale (logit), we summarized turnover effects as response‐scale average marginal effects (AMEs) computed from posterior draws. For each posterior draw, we evaluated the marginal effect of turnover on spatial stability for every site pair (given its observed environmental heterogeneity), converted effects to the response scale, and averaged across all pairs. We then quantified confounding bias as in Equation (3).

(3) $${\text{ΔAME}}_{\text{turnover}}={\mathrm{AME}}_{\text{full}}-{\mathrm{AME}}_{\text{reduced}}.$$

Positive ΔAME_turnover_ indicates that omitting environmental heterogeneity biases turnover effects downward.

#### Aggregation of simulation results

Each simulation of the metacommunity model incorporated stochastic variables, including random maximum growth rates *r*_*i*_, species environmental optima, interspecific competition coefficients, and dispersal influenced by inter‐site distances (Thompson et al., 2020). For each replicate, we fitted statistical models to the simulation output to estimate key coefficients. To account for variability across replicates and obtain robust parameter estimates, we pooled the posterior samples for these coefficients from all 100 simulation replicates within each scenario (as defined in Table 1). From these combined posterior distributions, we calculated the mean and 90% highest density interval (HDI) as the credible interval (CrI) for each coefficient, providing a comprehensive summary of the estimate and its associated uncertainty.

#### Sensitivity analyses

To address the difference in timescales between equilibrium‐like simulation end points and early successional empirical communities, we repeated the full analysis workflow at early simulation timesteps and tracked inferred turnover and interaction effects through time (Appendix S1: Figure S7A). To assess whether aggregating species abundances into a single functioning metric can influence inferred turnover effects when taxa differ strongly in biomass (as in our experiment; Appendix S1: Figure S6A), we recalculated site‐level functioning as a weighted sum of species abundances for two scenarios of mass ratios (species have equal biomass; the largest species has 10 times more biomass than the smallest species) and repeated the modeling workflow (Appendix S1: Figure S7B). For the experiment, we additionally quantified how the coverage of the panels filled over time (mixture panels) as an indicator of increasing space limitation later in succession (Appendix S1: Figure S6B).

## RESULTS

**FIGURE 5:**
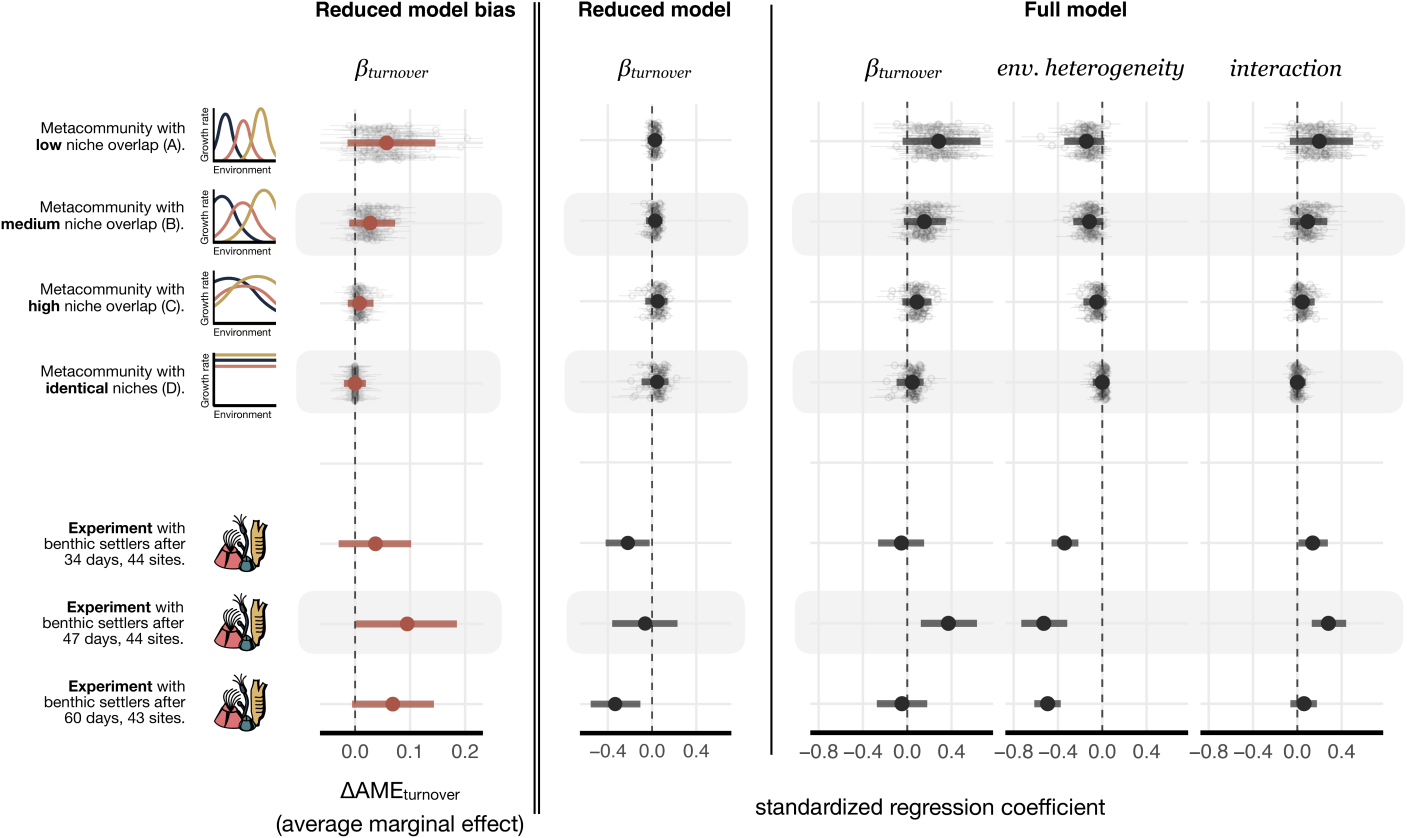
Confounded and corrected effects of species turnover on spatial biomass stability. Spatial stability was quantified as the ratio of total biomass between paired sites (min/max). Results are shown for simulated metacommunities across four niche‐overlap scenarios (Table 1; A: low overlap; B: medium overlap; C: high overlap; D: identical niches; *n* = 100 simulations per scenario) and for a field experiment sampled after 34, 47, and 60 days (*n* = 44, 44, and 43 sites). For each dataset, we fitted (i) a reduced (confounded) model including turnover only and (ii) a full model including turnover, environmental heterogeneity, and their interaction. Left (reduced model bias): bias attributable to omitting environmental heterogeneity, where AME is the response‐scale average marginal effect of turnover (expected change in spatial stability per +1 SD turnover, averaged over the observed distribution of environmental heterogeneity). Positive ΔAME_turnover_ indicates that the reduced model underestimates the turnover effect (downward confounding bias). Middle (reduced model): standardized turnover coefficient from the reduced model. Right (full model): standardized coefficients for turnover, environmental heterogeneity, and their interaction from the full model. Filled points show posterior means and horizontal bars show 90% credible intervals; vertical dashed lines indicate zero. For simulations, gray points show estimates from individual metacommunity replicates, and filled points summarize pooled effects across replicates. Illustrations by Maël Grosse.

In Figure 5, we examine the turnover–stability relationship in three complementary ways: The right panel shows the standardized regression coefficients from the full model (turnover, environmental heterogeneity, and their interaction). The middle panel shows what a reduced (confounded) model would suggest if environmental heterogeneity is omitted. Because turnover effects are context‐dependent in the full model (turnover × environmental heterogeneity) and coefficients are on the link scale (logit), the left panel summarizes the implied response‐scale confounding bias as difference in average marginal effect: ΔAME_turnover_ = AME_full_ − AME_reduced_. Across all simulated scenarios except complete niche overlap, and across all three experimental time points, ΔAME_turnover_ was positive, indicating downward bias (i.e., the reduced model tends to underestimate the stabilizing effect of turnover).

### Simulations

Across 100 simulations for each scenario (Table 1), we found that the spatially stabilizing effect of turnover was dependent on both niche overlap and environmental heterogeneity (Figure 5, top 4 rows). In scenarios with low niche overlap (Table 1, scenario A), we observed a positive interaction between turnover and environmental heterogeneity on biomass stability (mean aggregated coefficient: 0.20, 90% CrI [−0.07, 0.50]), alongside a positive main effect of turnover (0.28, 90% CrI [−0.04, 0.66]). This stabilizing interaction weakened progressively as niche overlap increased (scenario B: interaction 0.09 [−0.07, 0.27]; scenario C: 0.05 [−0.05, 0.16]). In the scenario with complete niche overlap (Table 1, scenario D), both the main effect of turnover (0.05, 90% CrI [−0.10, 0.15]) and the interaction with environmental heterogeneity (0.00, 90% CrI [−0.06, 0.08]) were negligible. Across scenarios A–C, environmental heterogeneity had a negative direct effect on stability (e.g., scenario A: −0.14, 90% CrI [−0.34, 0.02]), consistent with the idea that environmental differences create among‐site differences in productivity unless buffered by compositional turnover. Crucially, when environmental heterogeneity was omitted from the model (the reduced/confounded model), the inferred turnover effect was biased toward zero (and would miss the key interaction), particularly in the low and medium niche overlap scenarios. This is captured by the positive ΔAME_turnover_ values (Figure 5, left): scenario A ΔAME_turnover_ = 0.06 (90% CrI [−0.01, 0.15]; *p*(ΔAME > 0) = 0.90), scenario B = 0.03 ([−0.01, 0.07]; *p* = 0.88), scenario C = 0.01 ([−0.01, 0.03]; *p* = 0.72), whereas scenario D showed essentially no bias (ΔAME_turnover_ = 0.00 [−0.02, 0.02]; *p* = 0.49). Notably, the variation in effect sizes among individual metacommunity realizations was high (Figure 5, gray dots), underscoring the importance of aggregating results from multiple stochastic simulations. The source of this variation was mainly the different combinations of environmental optima, the randomized interspecific competition, and the randomized growth rates of the species in the simulation. The outcome was not sensitive to the sampling time (Appendix S1: Figure S7).

**FIGURE 6:**
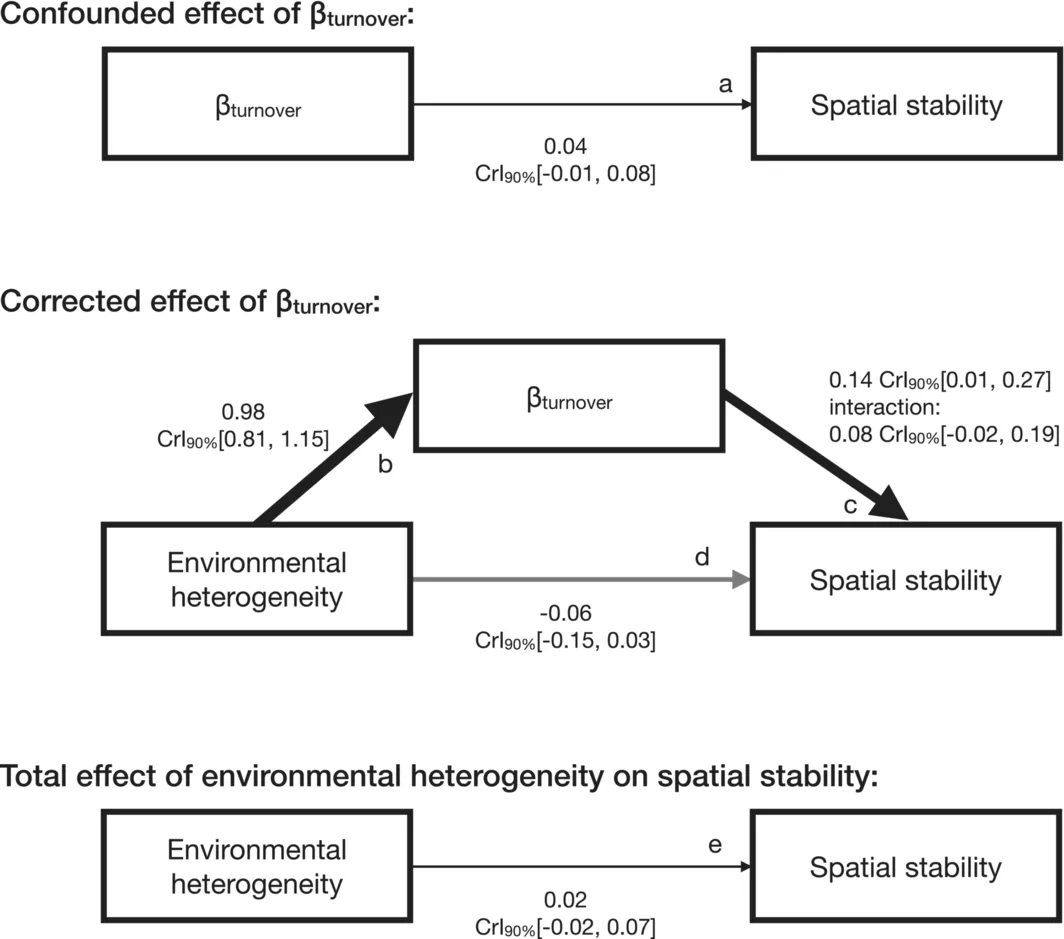
A mediation analysis on a single metacommunity model from scenario B, with medium niche overlap, reveals significantly different effects between the confounded effect of β‐diversity and the corrected effect of β‐diversity. The numbers represented are standardized regression coefficients. The size and color of the arrows indicate the strength and direction of the effect. The arrows depict (a) the confounded effect of turnover on biomass stability, which should not be interpreted, (b) the effect of environmental heterogeneity on turnover, (c) the effect of turnover on stability while correcting for environmental heterogeneity, (d) the direct effect of environmental heterogeneity on biomass stability, that is, not considering turnover, and (e) the total effect of environmental heterogeneity on biomass stability, including turnover.

To illustrate the causal pathways underlying these results, we examined a single representative metacommunity from the medium niche overlap scenario (Figure 6). In this iteration, the confounded effect of turnover on stability (effect a) was minimal (0.04, 90% CrI [−0.01, 0.08]). After accounting for environmental heterogeneity, the direct effect of turnover (effect c) became strongly positive (0.14, 90% CrI [0.01, 0.27]), accompanied by an additional interaction term (0.08, 90% CrI [−0.02, 0.19]). Environmental heterogeneity had a strong positive effect on turnover (effect b: 0.98, 90% CrI [0.81, 1.15]) but a negative direct effect on stability (effect d: −0.06, 90% CrI [−0.15, 0.03]). The resulting total effect of heterogeneity on stability was close to zero (0.02, 90% CrI [−0.02, 0.07]; effect e). This mediation analysis reveals how failing to account for the dual role of environmental heterogeneity, which both generates turnover and directly destabilizes biomass, can mask the true, positive contribution of turnover to spatial stability.

### Experiment

To evaluate whether the dominant taxa in our experiment with marine benthic communities differed in their environmental responses, we fitted generalized additive niche models for each OTU. These heatmaps (Appendix S1: Figure S2) show distinct biomass optima across the main environmental gradients (PC1 and PC2). Species having different environmental optima are a prerequisite for species sorting.

The communities showed the same qualitative pattern as the simulations: Turnover buffered the negative association between environmental heterogeneity and community stability (Figure 5, rows 5–7). Environmental heterogeneity had a consistently negative direct effect on stability at all time points (34 days: environmental heterogeneity = −0.34, 90% CrI [−0.46, −0.22]; 47 days: −0.53 [−0.73, −0.32]; 60 days: −0.49 [−0.61, −0.37]). At the same time, the turnover × heterogeneity interaction was positive at Days 34 and 47 (interaction = 0.14 [0.01, 0.28] and 0.28 [0.13, 0.44], respectively), and remained positive but more uncertain at Day 60 (0.06 [−0.06, 0.18]). The main effect of turnover was most clearly positive at Day 47 (turnover = 0.37 [0.12, 0.63]), whereas it was close to zero and uncertain at Days 34 and 60 (−0.05 [−0.26, 0.15] and −0.05 [−0.27, 0.18]).

The reduced/confounded model (omitting heterogeneity) would have suggested a neutral‐to‐negative effect of β‐diversity/turnover on stability (Figure 5, Reduced Model), including negative turnover effects at Days 34 and 60. This is where ΔAME_turnover_ is informative (Figure 5, left): ΔAME_turnover_ was positive at all three time points (34 days: 0.04, 90% CrI [−0.03, 0.10], *p*(ΔAME > 0) = 0.82; 47 days: 0.09 [0.00, 0.19], *p* = 0.95; 60 days: 0.07 [−0.01, 0.14], *p* = 0.93), indicating systematic downward bias when environmental heterogeneity is omitted.

Where the reduced model implied a negative turnover/β‐diversity effect (especially at 60 days), this is consistent with the mass‐disparity sensitivity analysis (Appendix S1: Figure S7B): When functioning is dominated by a small number of high‐biomass taxa (large mass ratios/strong dominance differences among sites), turnover can appear destabilizing even if this is not the turnover effect per se.

The weaker and more uncertain turnover effect at Day 60 coincided with a late‐successional phase in which communities approached panel “filling” (Appendix S1: Figure S6B). As open space on the panels became limited, between‐site differences in dominance structure increased, which can make turnover appear neutral or destabilizing even when the underlying turnover–heterogeneity interaction remains positive (Figure 5; Appendix S1: Figures S6B and S7B).

We analyzed the three time points separately because we were explicitly interested in spatial stability. Since we sampled destructively and measured environmental variables at each sample date, the three time points are independent. As predicted, the sites showed highly correlated changes in biomass over time (Appendix S1: Figure S3A), with a median pairwise site correlation of 0.89 (Appendix S1: Figure S3B). In addition, the median pairwise correlation of biomass over time demonstrates that biomass responses were highly synchronous among sites. This further justifies our explicit focus on spatial stability.

## DISCUSSION

By combining simulations with a field experiment, we show that species turnover can buffer the destabilizing effects of environmental heterogeneity on spatial stability. We also show that this stabilizing effect is easily obscured when heterogeneity is not explicitly accounted for. Our study provides strong evidence that species turnover stabilizes biomass production across heterogeneous landscapes, and that this effect is dependent on the strength of species sorting. We demonstrate that species turnover can buffer against the destabilizing effects of environmental heterogeneity, supporting the spatial insurance hypothesis (Loreau et al., 2003; van der Plas, 2019; Wilcox et al., 2017). Unlike some prior correlational studies, our causal framework explicitly separates the effects of turnover from those of environmental heterogeneity, resolving potential ambiguities in earlier work and advancing a mechanistic understanding of biodiversity–stability relationships. This separation is reflected directly in Figure 5 (left column): ΔAME_turnover_ is consistently positive (except under complete niche overlap), showing that omitting heterogeneity biases turnover effects downward.

The simulations revealed that the positive interaction between turnover and environmental heterogeneity on spatial stability (defined as the invariability of biomass) is strongest in scenarios with low to medium niche overlap (Table 1, Figure 5). This finding aligns with the theoretical predictions of the spatial insurance hypothesis, which posits that species' differential responses to environmental conditions contribute to maintaining ecosystem processes across multiple sites in a metacommunity (Loreau & de Mazancourt, 2013; Yachi & Loreau, 1999). Critically, this mechanism depends on the degree of niche overlap: When species have distinct environmental optima (low niche overlap), turnover reflects species sorting, which has a stabilizing effect on biomass production. As niches overlap completely, this insurance effect vanishes. Consistent with this, ΔAME_turnover_ is positive when niche overlap is low–medium but approaches zero under complete overlap, showing that confounding by heterogeneity is strongest when species sorting is strongest.

Our experiment broadly supports the findings from the simulations. We observed a positive interaction between turnover and environmental heterogeneity at all sampling dates, validating the theoretical predictions in a natural ecosystem. In addition, the main effect of turnover on stability was generally neutral to positive, although the uncertainty of these effects was higher in the experiment. Reduced models that omit heterogeneity can imply neutral‐to‐negative effects of species turnover (i.e., an apparent destabilizing β‐diversity effect), even when ΔAME_turnover_ indicates downward confounding bias. Where turnover appears destabilizing, this is consistent with our mass‐disparity sensitivity analysis, in which differences among species in biomass dominance can drive negative apparent turnover effects. The discrepancy between simulations and the experiment may also be attributed to priority effects, a phenomenon well documented in shallow marine benthic systems (Fraschetti et al., 2002; Irving et al., 2007; Vieira et al., 2018). Priority effects refer to the strong influence of early‐arriving species on community development. They can lead to alternative stable states and potentially alter the expected relationship between diversity and stability (Fukami, 2015). Within Vellend's (2016) framework, priority effects highlight the importance of historical contingency (see also Chase & Leibold, 2003), where the sequence of species arrival (dispersal) and initial establishment success (potentially involving ecological drift) shape subsequent community development, often leading to alternative states maintained by positive frequency‐dependent selection. In our study, priority effects could occur if barnacles or bryozoans dominated the panels early in the succession, preventing later‐arriving species from establishing. Specifically, such early dominance might have reduced the overall level of species turnover achieved during the experiment, potentially masking the full stabilizing effect of turnover on biomass production that was evident in the simulations where community assembly was more controlled. Overall, our results suggest that species turnover buffers the negative impact of environmental variability on the spatial stability of community biomass. However, the role of stochastic processes like priority effects in modulating this relationship in natural systems should not be overlooked.

The tendency for turnover effects to be strongest mid‐succession and weaker or more uncertain after 60 days is consistent with assembly dynamics in which space limitation and increasing dominance by a few taxa over time constrain further compositional change. As the coverage of panels approaches saturation (Appendix S1: Figure S6B), between‐site differences may reflect which taxa become dominant rather than ongoing niche‐based sorting, which can dampen or even reverse apparent turnover–stability relationships.

Our analysis underscores that controlling for environmental heterogeneity is essential when assessing the impact of β‐diversity on spatial stability. This is especially true if we assume large environmental variation between sites and differential responses of species to this variation (Stein et al., 2014). In our simulations, failure to account for heterogeneity led to a clear underestimation of the effect of turnover on spatial stability in low and medium niche overlap scenarios, and this bias was evident across all experimental time points. In contrast, this underestimation was not observed in high or full niche overlap simulations, where such bias is not expected. The discrepancy highlights the potential for omitted variable bias (Byrnes & Dee, 2025; Rinella et al., 2020) to contribute to inconsistent results in previous studies (van der Plas et al., 2023). Our comparison between the full model (including heterogeneity) and the confounded model (excluding it) starkly illustrates this point: Failing to account for heterogeneity can mask or even reverse the perceived effect of turnover, as visualized in our mediation analysis (Figure 6) and regression results (Figure 5). While many biodiversity–ecosystem functioning experiments have intentionally minimized environmental heterogeneity (Gonzalez et al., 2020), our results align with those of Daleo et al. (2023). By independently analyzing heterogeneous and homogeneous environments as categorical variables, they implicitly controlled for environmental heterogeneity, yielding results consistent with ours. In Daleo et al. (2023), β‐diversity had a destabilizing effect in homogeneous environments but no effect on stability in heterogeneous environments, a pattern that is consistent with our findings.

It is important to acknowledge the limitations of our study. While our simulations offered valuable theoretical insights, they only partially capture the dynamics of natural communities. Although no single metric can fully reflect every ecological function, our focus on biomass ratios naturally emphasizes only one dimension of ecosystem functioning. Moreover, quantifying environmental heterogeneity poses a considerable challenge; ideally, it should reflect the specific gradients to which species respond. In our experiment, the choice of environmental metrics was informed by prior knowledge of the system, and we applied a PCA to reduce the dimensionality of correlated variables and focus on the dominant environmental gradients. Consequently, restricting site comparisons to ecologically relevant spatial boundaries helps maintain comparable species pools and environmental conditions (Burley et al., 2016). Ultimately, more comprehensive studies across a variety of ecosystems are essential to clarify the wider applicability of our findings. A second limitation is that our simulations represent environmental heterogeneity along a single axis, whereas natural systems vary along multiple, partly correlated gradients. Multidimensional heterogeneity can increase opportunities for niche differentiation, but it can also introduce mismatches between the measured heterogeneity metric and the axes to which species actually respond. This may partly explain why experimental estimates were more uncertain than in simulations, despite broadly consistent directions of effect.

Determining species' niches and the degree to which they overlap is nontrivial, yet it is key to inferring the strength of species sorting and thus the underlying causes of turnover. The supplementary niche models (Appendix S1: Figure S2), although based on realized niches, illustrate that the focal taxa occupy distinct optima along the main environmental gradients, supporting this assumption in our study. The fundamental niche, which represents the range of environmental conditions a species can tolerate in the absence of competitors (Hutchinson, 1957), is often estimated under simplified conditions or from incomplete field data, leading to potential biases that may not reflect how a species actually performs under competition (Germain et al., 2018). Competition and other biotic interactions usually constrain species to their realized niches (Germain et al., 2018; Schrofner‐Brunner et al., 2023; Soberón & Arroyo‐Peña, 2017). When species share highly similar fundamental niches, the outcome of competition is harder to predict and turnover may not reflect species sorting, but rather ecological drift. In contrast, if species have different environmental optima, turnover is more likely linked to shifts in abiotic conditions. Recognizing these different pathways to turnover (and how they relate to niche overlap) is essential for interpreting how turnover affects spatial stability.

Spatial asynchrony (i.e., out‐of‐phase temporal fluctuations in biomass among local communities) is widely recognized as a key stabilizing factor in ecosystems (e.g., Qiao et al., 2023; Wang et al., 2021; Wilcox et al., 2017). Yet, spatial asynchrony does not directly address differences in mean functioning across sites. For example, two sites can show asynchronous biomass fluctuations (thus stabilizing aggregate biomass over time) yet differ substantially in their long‐term mean biomass due to environmental heterogeneity (Figure 2). However, low spatial asynchrony can also arise in systems with relatively short time windows or pronounced environmental gradients, where spatial differences in mean biomass exceed the magnitude of temporal fluctuations (Defriez & Reuman, 2017; Körner, 2007; Lubchenco, 1980; Sporn et al., 2010; van der Wal et al., 2010). Because our aim was to isolate purely spatial effects, and because species' biomasses increased relatively synchronously, we focused on spatial stability, quantified as the invariability of the average functioning across sites. This approach provides a complementary perspective to spatiotemporal studies and a more direct test of the insurance hypothesis in relation to spatial environmental heterogeneity (Loreau et al., 2003). Nevertheless, future research could benefit from integrating both spatial and temporal metrics to gain a more comprehensive understanding of how biodiversity mediates ecosystem functioning across scales. In particular, temporal environmental heterogeneity could interact with the spatial species sorting mechanism examined here: If the conditions favored at a site shift over time, the identity of the locally dominant species may track them so that the same sorting that evens out biomass across space could also drive compensatory dynamics through time.

Our study provides strong evidence for the spatially stabilizing effect of turnover in species composition on biomass production in heterogeneous environments. It also highlights the complexity of this relationship in natural systems. By demonstrating the importance of niche overlap, the causes of turnover, and controlling for environmental heterogeneity in analyses of β‐diversity effects, we contribute to resolving ongoing debates in the field (van der Plas et al., 2023) and provide a methodological framework for future research. Our framework involves (1) explicitly measuring and accounting for environmental heterogeneity across sites, (2) considering the potential role of niche overlap in mediating the effects of turnover, and (3) acknowledging the influence of stochastic processes. Moreover, these insights underscore the critical role of biodiversity in buffering ecosystem processes, providing practical guidance for management decisions such as establishing species‐specific niches in restoration efforts (Holl et al., 2022).

## AUTHOR CONTRIBUTIONS

Benedikt Brunner led the manuscript writing, with all authors contributing to revisions and comments. Benedikt Brunner, James G. Hagan, and Lars Gamfeldt conceptualized the study. Fieldwork was conducted by Benedikt Brunner, James G. Hagan, Julia Mari Murata, Lara Colaço Martins, Pedro de La Patelliere, and Elena Schrofner‐Brunner. Simulations and data analysis were performed by Benedikt Brunner, and data curation and analysis involved Benedikt Brunner, James G. Hagan, Julia Mari Murata, Lara Colaço Martins, Pedro de La Patelliere, and Jesper Hassellöv. All authors reviewed and approved the final manuscript.

## CONFLICT OF INTEREST STATEMENT

The authors declare no conflicts of interest.

## Supporting information


Appendix S1.

## Data Availability

Data and code (Brunner, 2026) are available in Zenodo at https://doi.org/10.5281/zenodo.20758643.
