# Supplementary material for: From environmental heterogeneity to stability: Beta‐diversity stabilizes biomass across space
Source: Ecology. 2026 Sep 17;107(9):e70498. doi: 10.1002/ecy.70498 (PMC13584661; doi:10.1002/ecy.70498)
Supplement: Supplementary file 1 [file ECY-107-e70498-s001.pdf]

Appendix S1

From environmental heterogeneity to stability: Beta-diversity stabilizes biomass across space

Benedikt Brunner, James G. Hagan, Jesper Hassellöv, Julia Mari Murata, Lara Colaço Martins, Pedro de La Patelliere, Elena Schrofner-Brunner, Lars Gamfeldt

Ecology

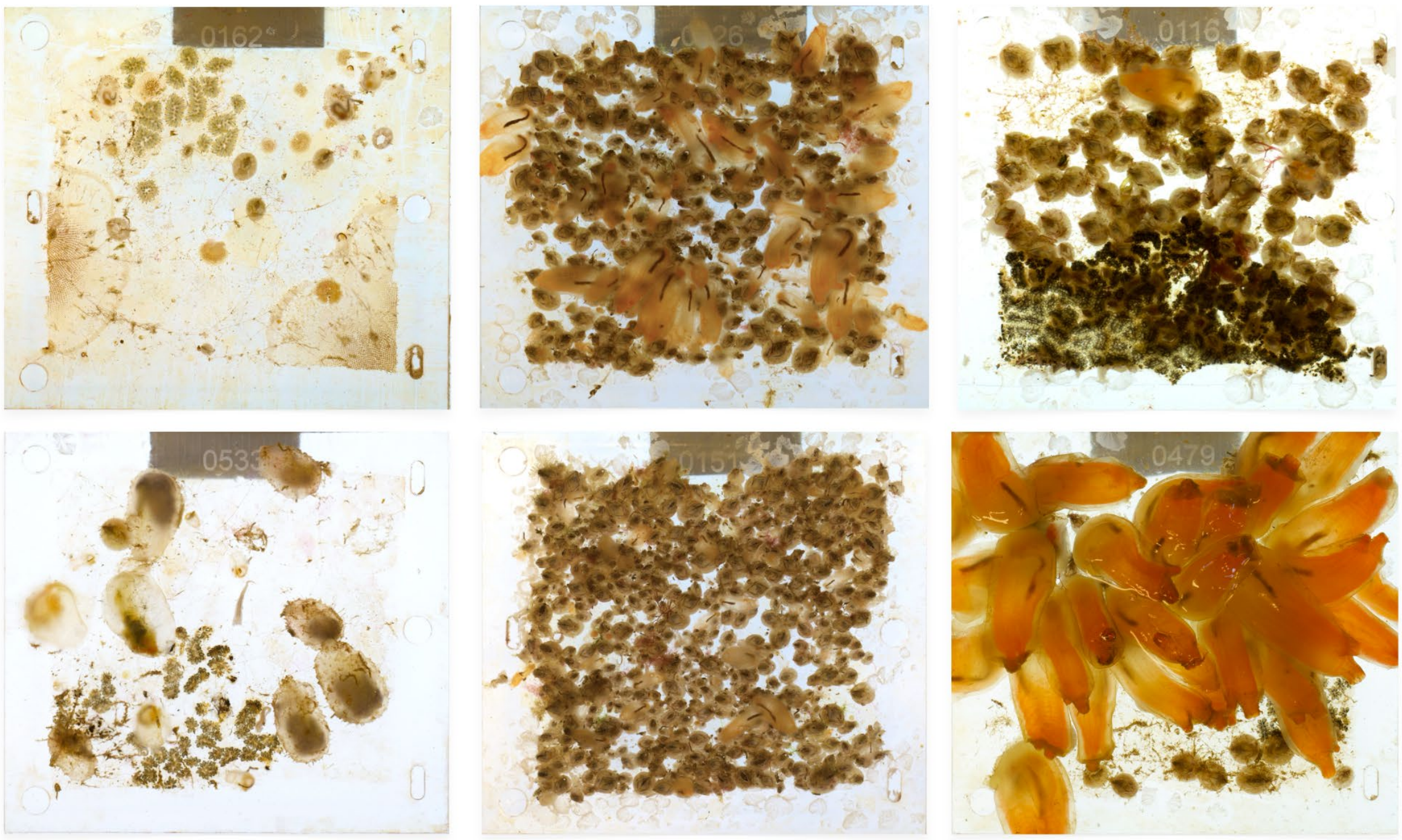

Figure S1: Example communities from different sites with species from the surrounding environment settling on PMMA panels: Ciona intestinalis, Ascidiella scabra, Amphibalanus improvises. Photographs by Benedikt Brunner.

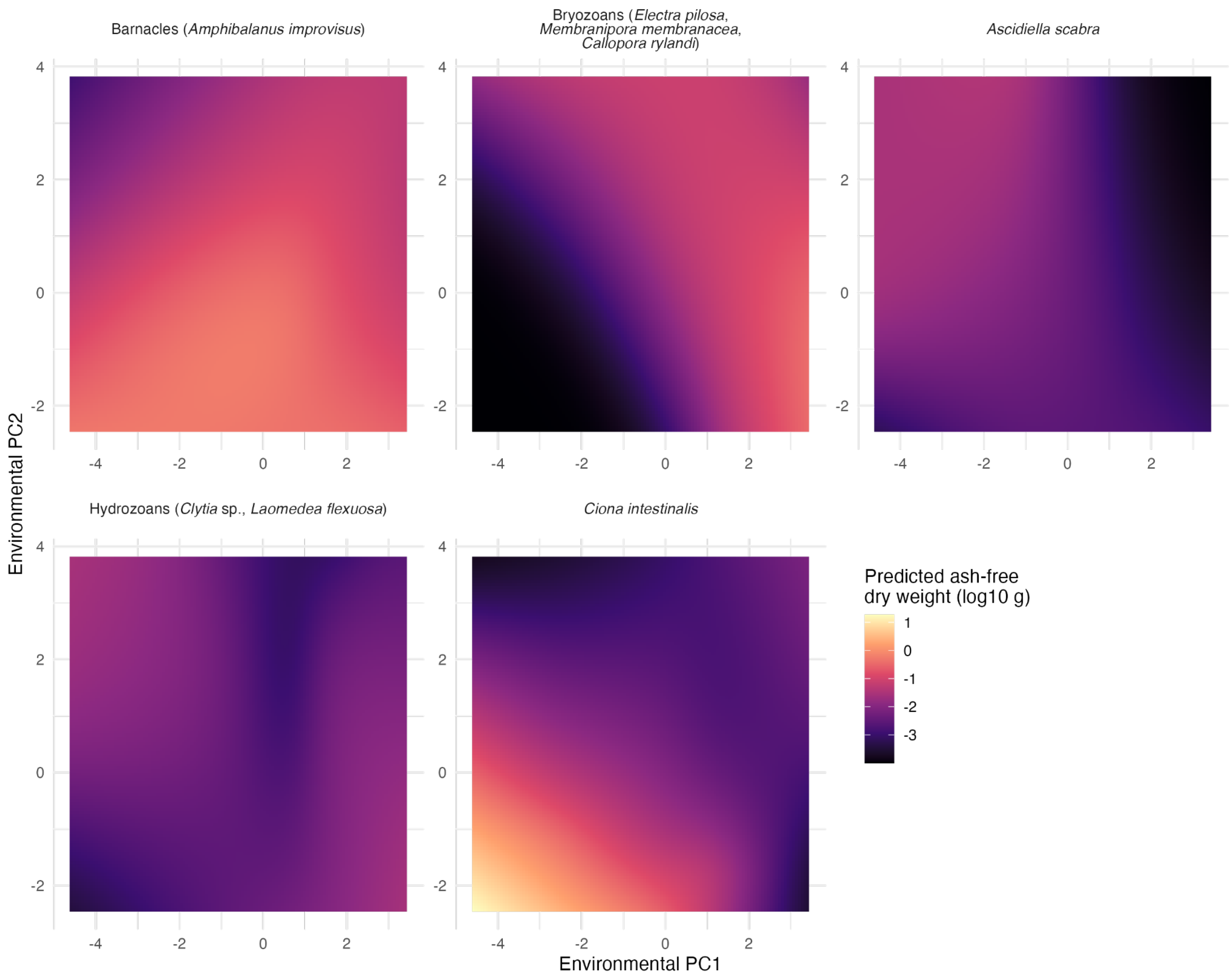

Figure S2: Generalised additive model (GAM) predictions of ash-free dryweight (g) across environmental gradients of PC1 and PC2, with PC3 held at its median. Panels show barnacles (Amphibalanus improvisus), bryozoans (Electra pilosa, Membranipora membranacea, Callopora rylandi), Ascidiella scabra, hydrozoans (Clytia sp., Laomedea flexuosa), and Ciona intestinalis. These niche models illustrate that the taxa occupy different environmental optima, consistent with species sorting along environmental gradients.

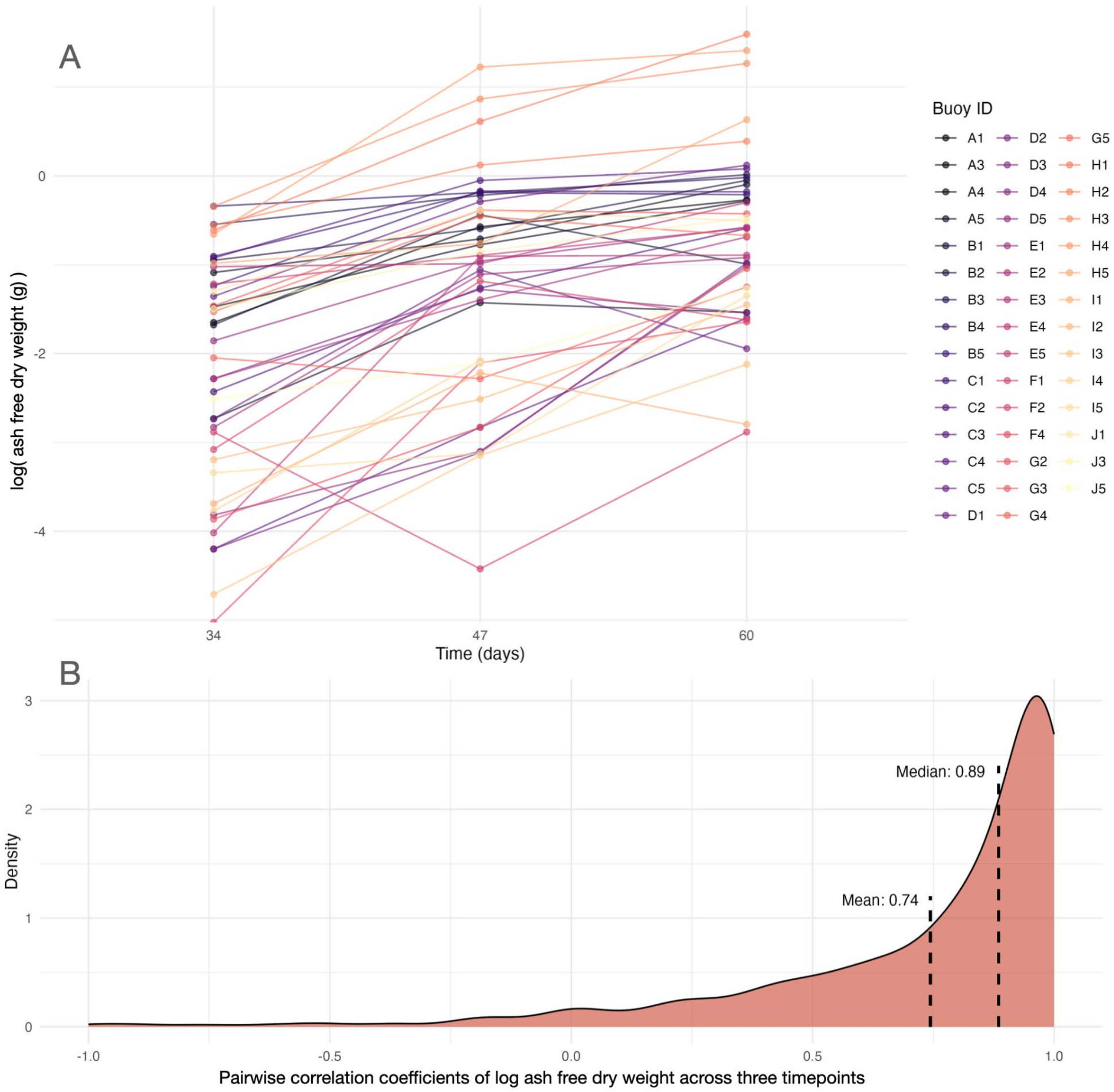

Figure S3: A: Log of ash-free dry weight (g) coloured by site (n=44). B: Density plot of pairwise (each site pair) correlation of ash-free dry weight over time as synchrony measure.

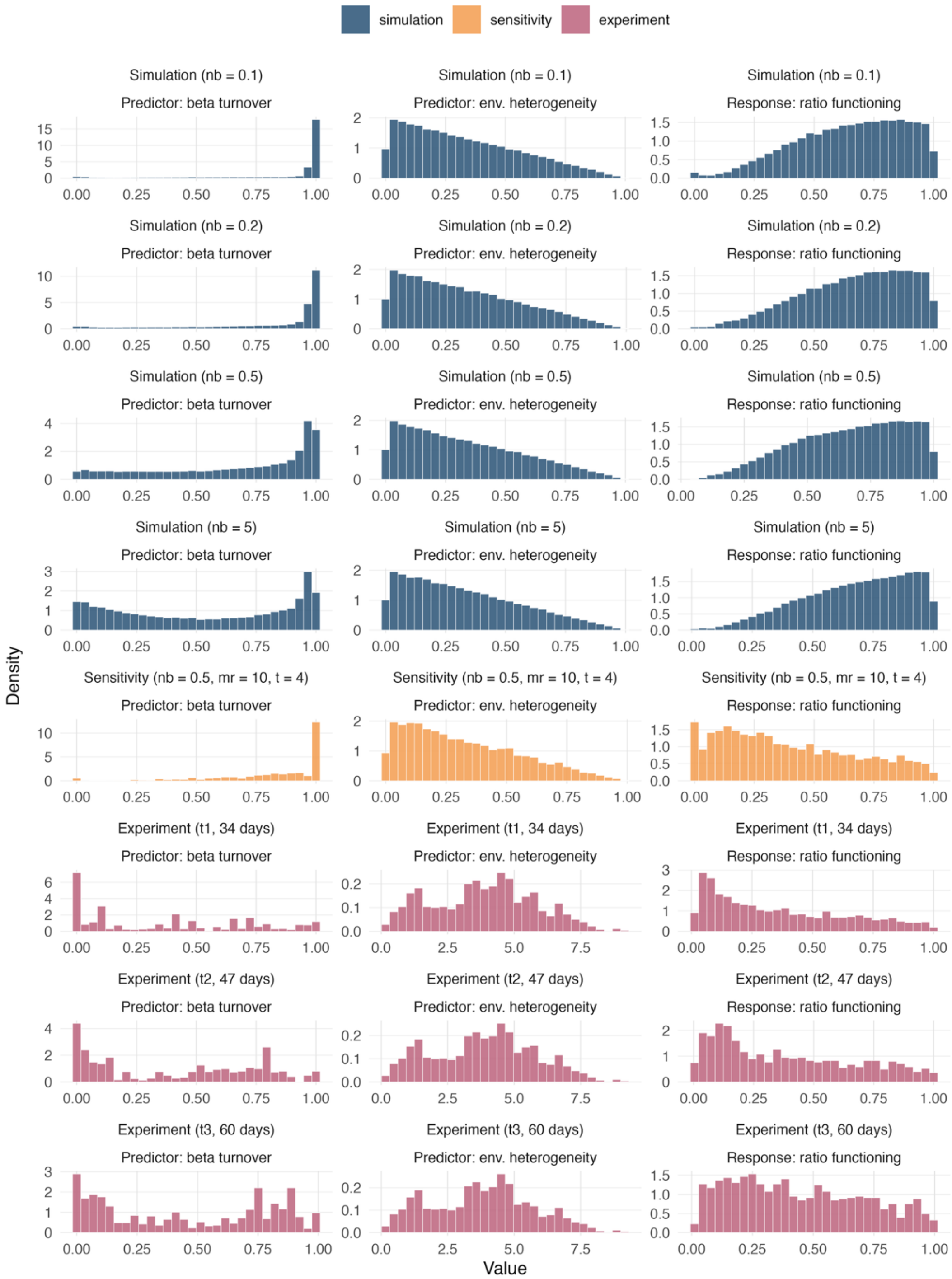

Figure S4. Distributions of predictor and response values used in simulation, sensitivity, and experimental datasets. Rows show simulation scenarios (niche breadth = 0.1, 0.2, 0.5, 5), one sensitivity setting (niche breadth = 0.5, mass ratio = 10), and experimental sampling times (t1 = 34 d, t2 = 47 d, t3 = 60 d); columns show beta turnover, environmental heterogeneity, and ratio functioning.

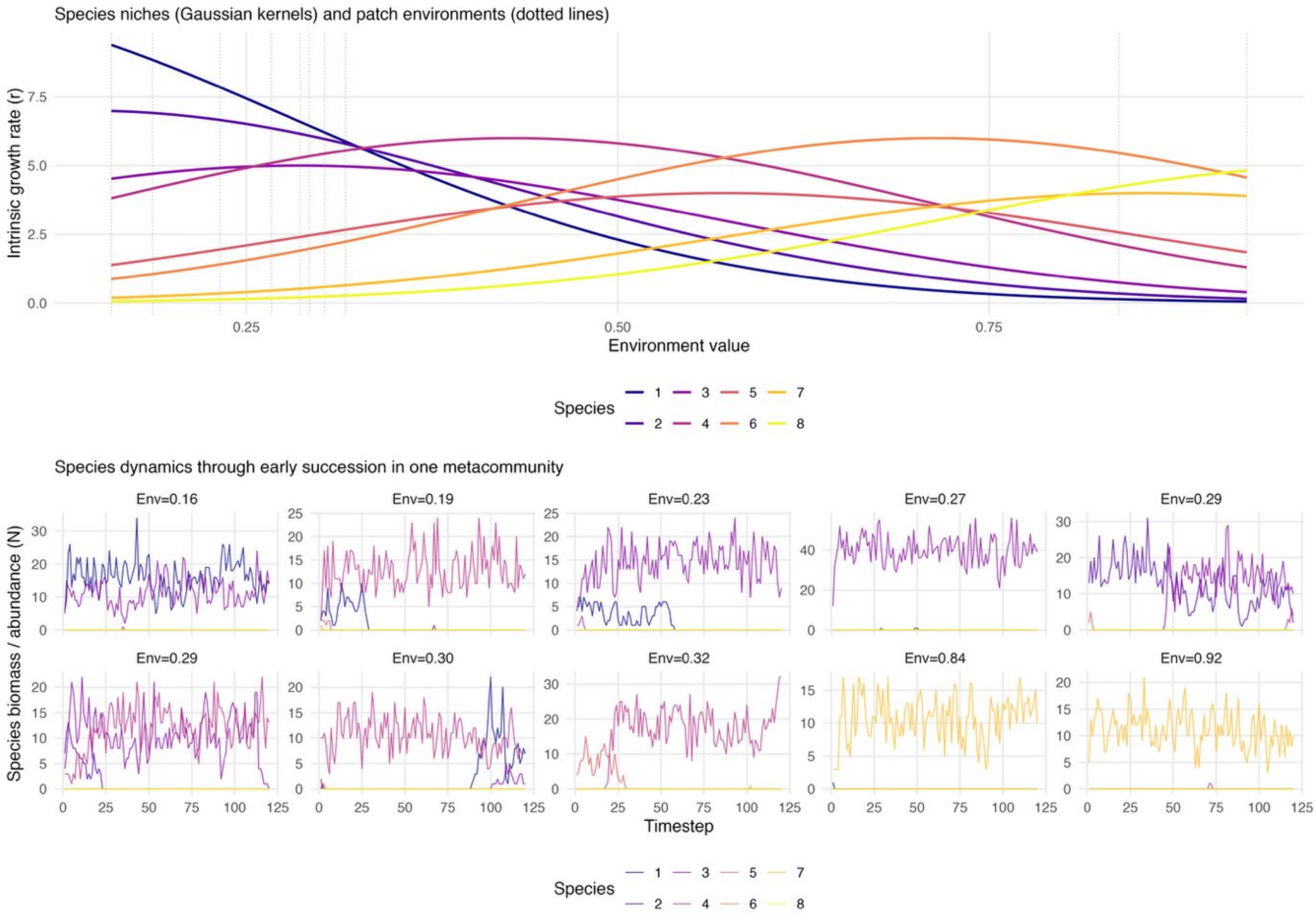

Figure S5. Example metacommunity showing species abiotic responses and transient dynamics across an environmental gradient. The top panel shows species intrinsic growth responses (Gaussian niches) with vertical lines marking site environment values. Lower panels show species abundance/biomass trajectories through time for sites spanning the gradient (environment value shown in each facet), illustrating species sorting and transient succession dynamics in the simulation model.

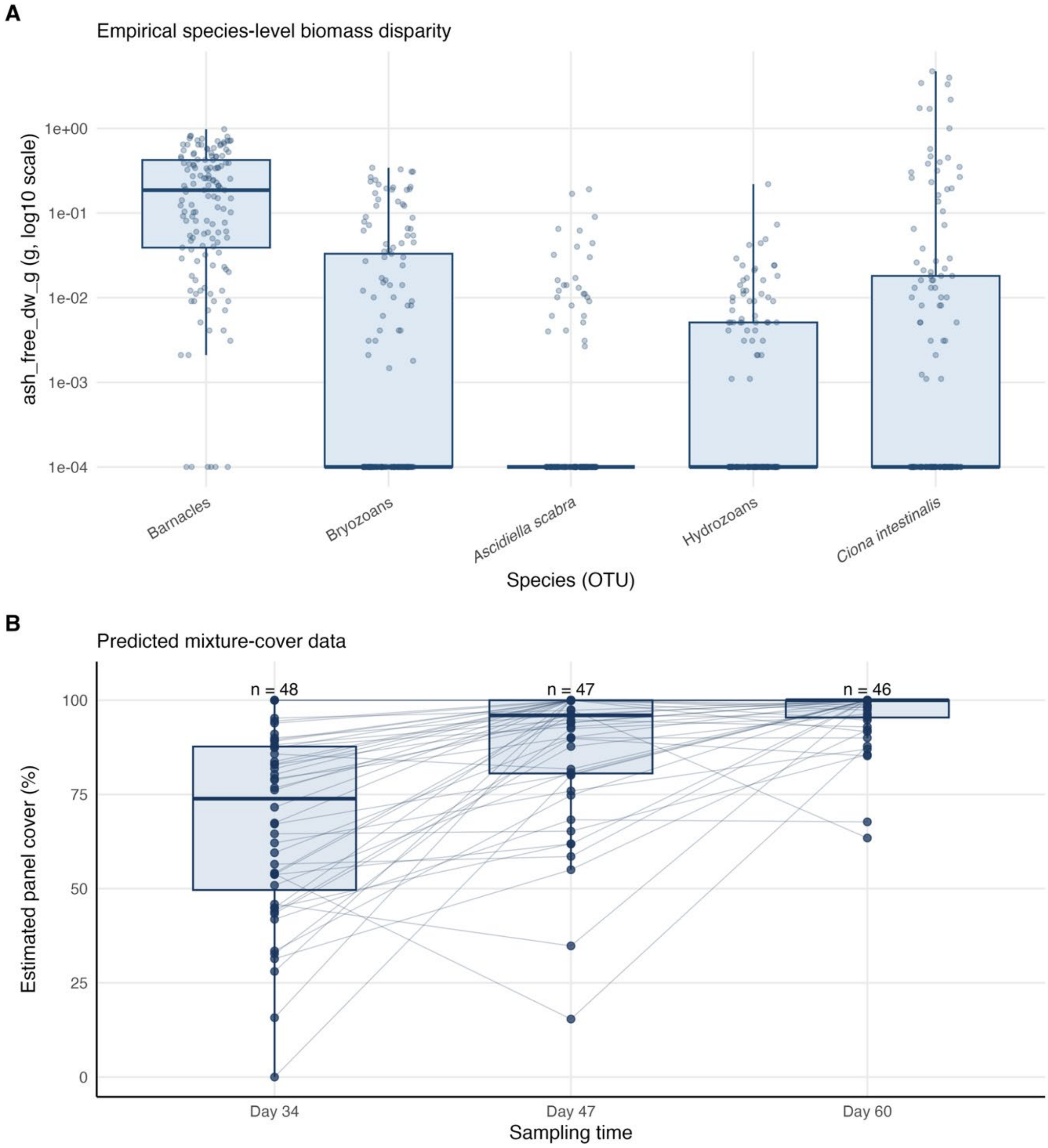

Figure S6. Biomass disparity among taxa and increasing panel filling over time in mixture panels. (A) Distributions of species-level ash-free dry weight (g; log10 scale) for each OTU (Barnacles, Bryozoans, Ascidiella scabra, Hydrozoans, Ciona intestinalis). Points show individual observations; boxplots show medians and interquartile ranges. (B) Estimated total cover (%) of mixture panels across sampling dates (sample sizes shown above each time point), with lines linking repeated panel series. Total cover was estimated from OTU-level proportional covers and approaches saturation by Day 60.

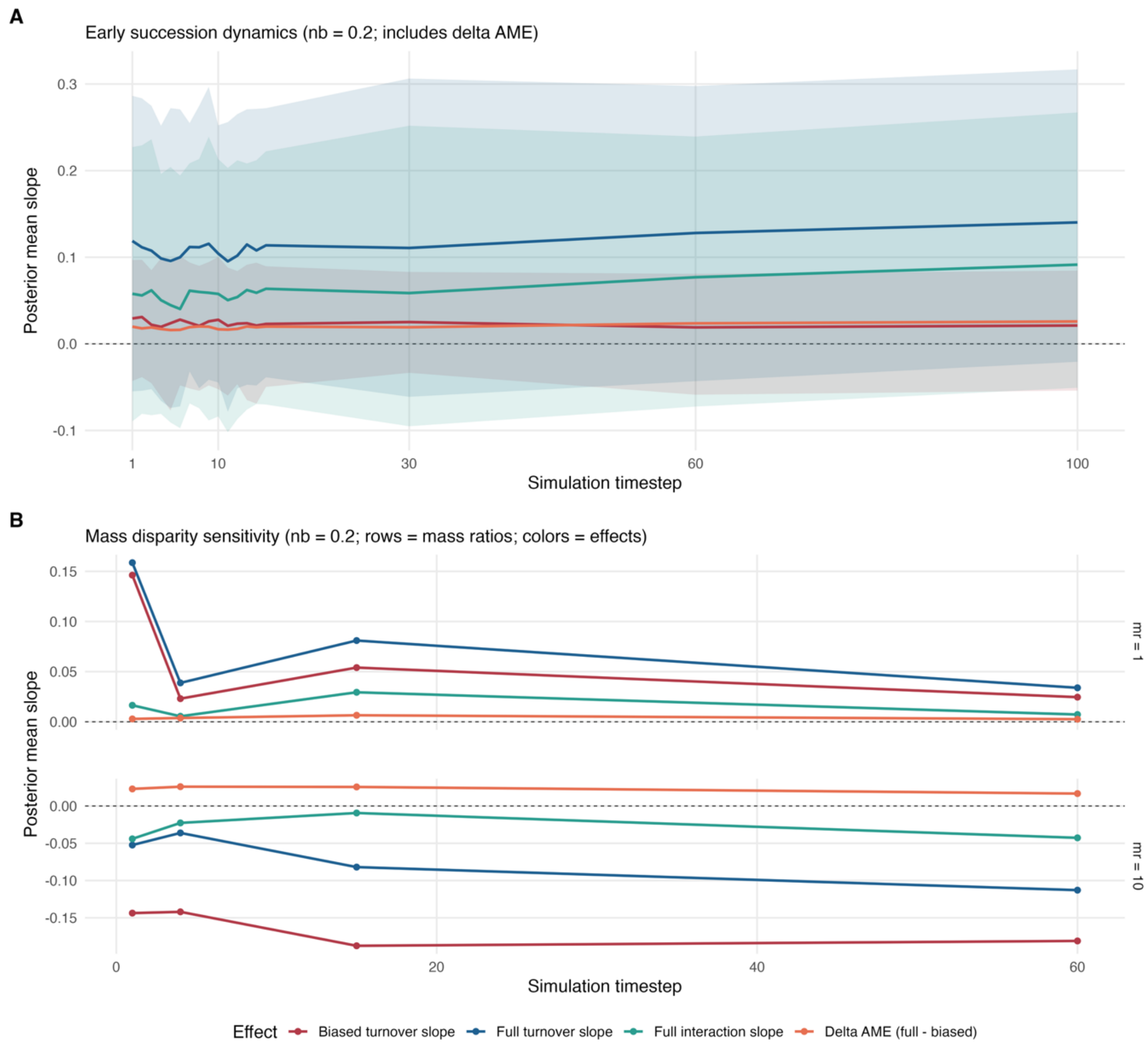

Figure S7. Sensitivity of inferred model effects to sampling time during succession and to mass disparity. (A) Early-succession dynamics for niche breadth = 0.2: posterior mean effects across simulation timesteps for the biased turnover slope, full-model turnover slope, full-model interaction slope, and delta AME (full - biased); shaded ribbons show 90% credible intervals where available. (B) Mass-disparity sensitivity for niche breadth = 0.2, shown for mass ratio = 1 and mass ratio = 10 (rows), with colours indicating effect type across panels. Here, mass ratio is the ratio of species weights used in the functioning metric (w_largest / w_smallest). Points/lines summarise posterior means at t = 1, 4, 15, 60 (n = 5 simulations per mass-ratio setting per timepoint). Dashed horizontal lines indicate zero.

Supplementary Methods

Section S1: Simulation framework

We simulated metacommunities using the discrete-time Beverton–Holt process-based metacommunity model described by Thompson et al. (2020), implemented with *mcomsimr* (`simulate_MC`) and project-specific wrapper scripts. In brief, each timestep combines: (i) density-independent abiotic responses (Gaussian niches) to the site environment, (ii) local density dependence via a competition matrix, (iii) demographic stochasticity, and (iv) dispersal among sites with distance decay. A small extirpation probability is applied to prevent indefinite persistence of extremely rare populations. The repository/Zenodo archive documents the exact implementation and parameter values used.

Model specification

For species i in site x at time t, local dynamics follow

$$N_{i,x}(t+1) = \frac{N_{i,x}(t)\, r_{i,x}}{1 + \sum_{j=1}^{S} \alpha_{ij}\, N_{j,x}(t)} + I_{i,x}(t) - E_{i,x}(t),$$

where $r_{i,x}$ is the density-independent abiotic response, α_{ij} are competition coefficients, and $I_{i,x}(t)$ and $E_{i,x}(t)$ are immigration and emigration terms, respectively.
Abiotic responses are Gaussian niches on the site environment E_x:

$$r_{i,x} = r_{\max,i} \exp\left(-\frac{(E_x - \theta_i)^2}{2\sigma^2}\right),$$

where θ_i is the environmental optimum and σ controls niche breadth (larger σ implies higher niche overlap).
Emigration is proportional to local abundance,

$$E_{i,x}(t) = m\, N_{i,x}(t),$$

with m the emigration rate. Immigration redistributes emigrants among sites using a distance-decay kernel $w_{y \to x}$ that is normalised such that $\sum_x w_{y \to x} = 1$ for each source site y:

$$I_{i,x}(t) = \sum_{y \neq x} E_{i,y}(t)\, w_{y \to x}$$

This kernel is exponential, $w_{y \to x} \propto exp(-\kappa\, d_{xy})$, where d_{xy} is the distance between sites x and y and the kernel exponent κ sets the rate of distance decay (larger κ produces more spatially restricted dispersal); inter-site distances are measured on a torus to avoid edge effects (the *mcomsimr* default; Thompson et al. 2020). Parameter values, including κ, are given in Table S1.

Section S2: Simulation parameterisation

Table S1 summarises parameter settings for baseline simulations used in the main text and for transient sensitivity runs. For the mass-disparity sensitivity, "mass ratio" (mr) denotes the ratio of species weights used in the functioning metric ($w_{\text{largest}}/w_{\text{smallest}}$).

Parameter	Legacy main runs	Early succession effects (panel A)	Mass-disparity (panel B)
Species (S)	20	20	20
Sites (P)	50	50	50
Niche breadth (σ)	0.1, 0.2, 0.5, 5.0	0.2	0.2 (and 0.5 in grid)
Analysed timesteps	100	100	60
Burn-in	100	20	0
Initialisation	100	20	0
Emigration (m)	0.01	0.01	0.001
Kernel exponent (κ)	0.1	0.1	0.1
Intraspecific competition	1	1	1
Interspecific competition (pre-scale)	Uniform(0, 1)	same	same
Competition scaler	0.05	0.05	0.25
Extirpation probability	1×10^{-5}	1×10^{-5}	1×10^{-5}
rmax,s	Poisson(5), species-specific	same	same
Optima spacing (θ)	even over [0,1]	even	even
Environment (E)	site-level U(0,1), constant in time	same	same
Functioning weights	unweighted	unweighted	bimodal (mr); 25% spp. at mr, remainder at 1

Section S3: Sampling design and time-scale sensitivity

Sampling design differed by analysis objective. Baseline ("legacy") simulations were run with an initialisation phase (100 timesteps) and a burn-in phase (100 timesteps), followed by 100 analysed timesteps (total loop length 300); main-text analyses use the final analysed timestep. The initialisation phase corresponds to the colonisation (community assembly) phase, during which species establish and sort across the landscape from the initial seeding; the subsequent burn-in phase then allows the assembled metacommunity to approach a stable equilibrium before the analysed timesteps are sampled.

For transient succession sensitivity, simulations used shorter initialisation/burn-in (20/20 timesteps) and were sampled densely early (t = 1–15) with additional reference points (t = 30, 60, 100). Mass-disparity transient runs targeted early succession by omitting initialisation and burn-in (0/0 timesteps) and sampling at t = 1, 4, 15, and 60. These designs explicitly evaluate sensitivity to sampling time rather than assuming equilibrium.

Section S4: Average marginal effects (AME) and confounding bias (ΔAME)

Because turnover effects are context-dependent in the full model (turnover × environmental heterogeneity) and coefficients are estimated on the link scale, we summarised turnover effects as response-scale average marginal effects (AMEs) computed from posterior draws. For each dataset, we computed the marginal effect of turnover on spatial stability for each site pair (given its observed environmental heterogeneity) and averaged across pairs. Confounding bias was quantified as

$$\Delta AME_{\text{turnover}} = AME_{\text{full}} - AME_{\text{reduced}},$$

where the reduced model omits environmental heterogeneity. Positive $\Delta AME_{\text{turnover}}$ indicates that omitting heterogeneity biases the inferred turnover effect downward. Implementation details are provided in the code archive.

Section S5: Sensitivity analyses and empirical succession context

Early-succession sensitivity: We repeated the full pairwise workflow across multiple simulation timesteps (Section S3) to track how inferred turnover and interaction effects change through assembly.

Mass-disparity sensitivity: The relationship between species turnover and spatial stability may be affected by relative biomass (mass ratio) of the species. To test this, we recomputed site-level functioning as a weighted sum of species abundances using a bimodal weighting scheme (Table S1) and re-ran the workflow. We recalculated site-level functioning as a weighted sum of species abundances for two scenarios of mass ratios (species have equal biomass; the largest species has 10 times more biomass than the smallest species).

Empirical panel filling: to contextualise late-successional dynamics, we summarised increasing space pre-emption in mixture panels over time using an estimated total cover metric derived from OTU-level covers (details and results shown in the Supplementary Figures).

Section S6: Software, priors, and reproducibility

All analyses were conducted in R; key packages included *brms* (Bayesian Beta-family mixed models), *cmdstanr*/Stan, *mcomsimr*, *betapart*, and *vegan* (full session information in the repository/Zenodo archive). Predictors were standardised (z-transformed). We did not specify custom priors; models, therefore, used the default *brms* priors for the corresponding model classes. Stan control settings (e.g., `adapt_delta`, `max_treedepth`) and convergence diagnostics (R-hat, effective sample size, divergences) are archived with the analysis scripts.

References

Thompson, P. L., L. M. Guzman, L. De Meester, Z. Horváth, R. Ptacnik, B. Vanschoenwinkel, D. S. Viana, and J. M. Chase. 2020. A process-based metacommunity framework linking local and regional scale community ecology. Ecology Letters 23:1314–1329.
